# MicroRNAs Expression in the Ileal Pouch of Patients with Ulcerative Colitis Is Robustly Up-Regulated and Correlates with Disease Phenotypes

**DOI:** 10.1371/journal.pone.0159956

**Published:** 2016-08-18

**Authors:** Shay Ben-Shachar, Henit Yanai, Hadas Sherman Horev, Hofit Elad, Liran Baram, Ofer Issakov, Hagit Tulchinsky, Metsada Pasmanik-Chor, Noam Shomron, Iris Dotan

**Affiliations:** 1 Genetic Institute, Tel Aviv Medical Center, Tel Aviv, Israel; 2 IBD Center, Department of Gastroenterology and Liver Diseases Tel Aviv Medical Center, Tel Aviv, Israel; 3 Sackler Faculty of Medicine, Tel Aviv University, Tel Aviv, Israel; 4 Colorectal Unit, Division of Surgery, Tel Aviv Medical Center, Tel Aviv, Israel; 5 Bioinformatics Unit, G.S.W. Faculty of Life Sciences, Tel Aviv University, Tel Aviv, Israel; Laikon Hospital, GREECE

## Abstract

**Background:**

Gene expression alterations are associated with disease behavior in inflammatory bowel disease (IBD). microRNAs (miRNAs) are dominant in the regulation of gene expression, and may affect IBD phenotype. Our aim was to assess mucosal miRNA expression in IBD and the correlation with intestinal inflammation.

**Methods:**

We performed a large-scale analysis of ileal mucosal miRNA. Biopsies were retrieved from patients with ileal Crohn’s disease (CD), unoperated ulcerative colitis (UC) patients, UC patients after pouch surgery, and normal controls (NC). Pouch UC patients were classified as having a normal pouch (NP), chronic pouchitis (CP), and Crohn’s-like disease of the pouch (CLDP). miRNA expression was analyzed by parallel massive (next-generation) sequencing (NGS). Bioinformatics tools were applied for clustering and the detection of potential targets.

**Results:**

Sixty-one subjects were recruited. The ileum of unoperated UC patients was comparable with NC. There were significant miRNA expression alterations (fold change ≥2, corrected *P* ≤.05) in NP (n = 6), CP (n = 40) and CLDP (n = 139), but only two expression alterations were noted in CD. More than 90% of the altered miRNAs were up-regulated, and many were predicted to be associated with significantly decreased transcripts. miRNAs alterations were generally clustered with disease phenotypes.

**Conclusions:**

Ileal inflammation causes increased miRNA expression. miRNA alterations correlate with IBD phenotype, apparently by controlling the down-regulation of specific mRNAs.

## Introduction

The etiology of inflammatory bowel diseases (IBD), i.e. Crohn’s disease (CD) and ulcerative colitis (UC) remains unknown. CD may affect any part of the gastrointestinal tract, while UC affects only the colonic mucosa. Up to one-quarter of all UC patients require colectomy due to intractable disease or its complications.[1] That procedure, restorative proctocolectomy with an ileal pouch-anal anastomosis (IPAA), involves resection of the entire large bowel and use of the unaffected small bowel to create a reservoir (pouch) connected to the anal canal.[2–4] IPAA is the surgery of choice for refractory UC,[5–9] but it has a substantial rate of complications.[10, 11] Pouch inflammation (“pouchitis”) is the most prevalent long-term complication, affecting more than 50% of those patients.[7] Pouchitis may have different disease behavior patterns: the acute form is defined by flares that resolve after short-term antibiotic treatment, recurrent acute pouchitis is defined as ≤4 flares within one year with periods of remission between them, and chronic pouchitis (CP) is characterized by more than 4 weeks of symptoms necessitating prolonged antibiotic or anti-inflammatory therapy, or by ≥5 flares of recurrent acute pouchitis/year. Some patients may develop a form of chronic inflammation that resembles CD, with fistulas, strictures, or inflammation of ileal segments proximal to the pouch; this form is termed Crohn’s-like disease of the pouch (CLDP).[12–17]

Because the inflammation in UC is characteristically restricted to the colon, the development of inflammation in the ileal pouch is considered an unexpected event. We had previously shown that messenger RNA (mRNA) expression profiles correlate with disease behavior and the degree of inflammation in ileal IBD.[18] MicroRNAs (miRNAs) are endogenous non-coding RNAs, approximately 22 bases in size. miRNAs play important gene-regulatory roles in living organisms by pairing with the mRNAs of protein-coding genes to direct their post-transcriptional repression.[19, 20]. Hundreds of miRNAs are known to exist in humans, and each can modulate the expression levels of multiple distinct mRNA targets by specific hybridization to the 3’ untranslated region (3’UTR) of the mRNA. Data on the key role played by miRNA in human diseases, including tumorigenesis, cardiovascular and neurological diseases, have emerged in recent years.[21] Some miRNAs have been found to regulate or be regulated by factors already known to be associated with IBD pathogenesis, such as the microbiome, IL-12/IL-23p40 and NOD2.[22–24] It is therefore not surprising that a number of studies reported specific miRNA expression in IBD patients. Experiments using more robust screening methods, such as microarray analysis, identified a relatively small number of miRNAs that were inconsistently associated with IBD.[25–30]

We had previously shown that alterations in gene expression in pouchitis are more robust than in CD. We have further hypothesized that using pouchitis as a model for ileal IBD may enable the detection of larger numbers of miRNAs associated with intestinal inflammation. The current work aimed to compare autologous miRNA and mRNA alterations in order to evaluate possible associations between miRNAs and alterations in gene expression in IBD that may allow for a more comprehensive understanding of the disease and its etiology. We have also evaluated the effect of the inflammatory process on miRNA expression. For the first time, this study demonstrates that multiple miRNAs are altered in pouchitis in correspondence with the degree of inflammation. Moreover, more than 90% of the altered miRNAs were up-regulated, and many of those were predicted *in silico* to be associated with significantly decreased mRNA transcripts.

## Materials and Methods

### Patients and Controls

IBD (CD, unoperated UC and operated UC) patients were prospectively recruited at a tertiary IBD Center. CD and UC were defined according to accepted clinical, endoscopic, histologic, and imaging studies. Further inclusion criteria were the presence of ileitis for CD patients and having a normal terminal ileum as seen by endoscopy and confirmed by histology for unoperated UC patients. Pouch patients were further stratified into three groups, as previously described: normal pouch (NP), chronic pouchitis (CP), and Crohn’s-like disease of the pouch (CLDP).[7, 18, 31] Briefly, an NP was defined as being asymptomatic, having a pouchitis disease activity index (PDAI) score of ≤7 and being off medication for at least 24 months.[32] Patients with either an active flare longer than 4 weeks, with ≥5 flares/year or being treated with antibiotics or anti-inflammatory therapy for more than 4 weeks were classified as having CP. The criteria for having CLDP were having one or more of the following: pouch-related fistula, inflammation of the afferent limb or more proximal small bowel segment(s), and fibrostenotic disease of the pouch. Patients with acute or recurrent acute pouchitis were excluded from the study in order to prevent potential classification overlap. Individuals who required a colonoscopy for various indication(s), i.e., screening or evaluation of functional bowel disease with a normal ileal mucosa (by endoscopy and histology) volunteered to serve as normal controls (NC). The study was approved by the Tel Aviv Medical Center Institutional Review Board (IRB), approval number 0467–10 and by the Israeli national IRB committee number 0587-10/. Participants provided written informed consent.

### Clinical Data and Tissue Collection

Data on selected demographics, clinical variables, time from IBD diagnosis to proctocolectomy and IPAA, as well as pouch age (time from closure of ileostomy, where applicable, or from pouch surgery) were retrieved from the patients’ medical records. Biopsies were taken from the terminal ileum of the unoperated groups. Biopsies from pouch patients were taken by a single endoscopist (I.D.) from the most affected part within the pouch or from the middle of the pouch if there was no evidence of pathology. The biopsies were snap frozen and kept at -80°C until RNA extraction. Scoring of histopathologic slides according to the histology sub-score of the PDAI was performed by a single IBD-trained pathologist (EB). Briefly, active inflammatory characteristics were scored according to neutrophil infiltration (0–3) and area of ulceration (0–3). Infectious or mechanical etiologies for pouch inflammation were excluded using microbiological stool assessment and endoscopy, respectively.

### RNA Extraction

Total RNA was extracted from frozen biopsies using Trizol^®^ (Invitrogen, Carlsbad, CA) according to a standard protocol. Sample quality was assessed by both a photospectrometer (Nano Drop Technologies^®^, Thermo Scientific, Wilmington, DE) and agarose gels (1%). The 260/280 ratios in all samples were >1.8 and the 260/230 ratios were >1.7

### Massive Parallel RNA Sequencing (RNAseq)

Massive parallel (next-generation) sequencing (NGS) was carried out according to Illumina’s Small RNA sample preparation protocol (TruSeq Small RNA Sample Prep Kits, Illumina, San Diego, CA) for generating small RNA libraries directly from total RNA and for sequencing. miRNA libraries were sequenced on the HiSeq 2500 (Illumina) following the manufacturer’s protocol.

### Quantitative RT-PCR (qRT-PCR)

cDNAs were generated using 10 ng of RNA and specific primers (Life Technologies, Carlsbad, CA) according to the manufacturer’s instructions. qRT-PCR reactions were performed using TaqMan chemistry and the ABI 96-well platform (Life Technologies). Transcripts were tested, analyzed and normalized relative to housekeeping miRNAs (RNU24, and U6 using ExpressionSuite^®^ v1.0.3 software, Life Technologies).

### Tissue Culture

The human intestinal epithelial cell lines Caco-2 and HCT-116 were purchased from the American Type Culture Collection (ATCC, Manassas, VA, USA). Cells were maintained in supplemented EMEM (Caco-2) or RPMI 1640 (HCT116) (both from Biological Industries, Beit HaEmek, Israel), containing 10% FBS, 100 U/ml penicillin and 100 mg/ml streptomycin in a humidified atmosphere of 95% air, 5% CO_2_ at 37°C. Stimulation with inflammatory cytokines was conducted on 80% confluent cells that were incubated for 6 hours (without serum) with tumor necrosis factor alpha (TNF-α), interleukin 1 beta (IL-1β) and interferon gamma (INF-γ) (PeproTech, Rocky Hill, NJ, USA) at 10 ng/ml each.

### Statistical Methods and Bioinformatics

Statistical analysis for demographic characteristics was performed by SPSS software version 21 (SPSS Inc., Chicago, IL, USA). Continuous variables were compared by one-way analysis of variance and the non-parametric Kruskal-Wallis test. Categorical variables were explored by Pearson’s chi-square test. P values ≤0.05 were considered significant. Alterations in miRNAs expression were defined using a fold-change cutoff of >± 2.0, and a linear step-up false detection rate (FDR) of P ≤0.05 for the T-statistic, corresponding to the linear contrast comparing each group to the NC. Cluster analysis of differentially expressed genes was visualized by the Partek Genomics Suite using Pearson’s dissimilarity correlation and average linkage method. Post-processing analysis of significant transcripts was performed to determine a possible association between miRNAs and mRNA using the TargetScan database (release 6.2, 2012; http://www.targetscan.org/;[33]). miRNA lists were compared using Venn diagrams (http://www.cmbi.ru.nl/cdd/biovenn/;[34]). Association between miRNAs genomic location and coding genes location was calculated using the CoGeMir database (http://cogemir.tigem.it/;[35]). qRT-PCR validation was performed using DataAssist v3.0 software (Life Technologies). One-way analysis of variance with P values adjusted using the Benjamini-Hochberg FDR was performed. A P value <0.05 was considered significant.

## Results

Forty-nine IBD patients and 12 NC were recruited (Table 1). Patients with NP were significantly older compared to all the other groups: 53 years vs. 35–43 years (P = 0.038). Similarly, IBD duration (time from IBD diagnosis to study recruitment) was longer in the NP group compared to the CP and CLDP groups, as was the time between IBD diagnosis to pouch surgery. Altogether, this suggests that patients who do not develop pouchitis during the first years after surgery usually maintain a sustainable NP. IBD duration was the shortest for individuals with ileal CD. In the operated UC group, pre-operative exposure to steroids was noted in most (23/28, 82%) patients in contrast to a minority (3/28 10.7%) who were ineffectively exposed to anti-TNFs (Table 1).

**Table 1:** Demographics and clinical characteristics.

	Normal pouch (n = 12)	Chronic pouchitis (n = 12)	Crohn’s-like disease of the pouch (n = 4)	Ileal Crohn’s disease (n = 10)	Normal controls (n = 12)	Ulcerative colitis (n = 11)	P value
Gender (F/M)	5/7	5/7	2/2	6/4	7/5	3/8	NS^*^
Age at sampling, years, mean ±SD (range)	53.3±11.2 (34–66)	42.9±15.7 (18–71)	41.2±15.7 (24–56)	34.5±11.7 (20–51)	37.3±14.5 (20–64)	39.0±14.4 (21–69)	0.038^†^
Age at IBD diagnosis, years, mean ±SD (range)	23.6±9.9 (15–49)	23.2±12.3 (11–53)	28.2±14.6 (16–45)	29.5±13.4 (15–49)	NA	28.6±13.9 (13–56)	NS^†^
IBD duration, years, mean ±SD (range)	30.2±11.2 (9–45)	20.4±9.9 (6–37)	14.0±4.2 (9–18)	6.0±4.5 (1–12)	NA	11.36±7.9 (4–30)	>0.001^†^
Time between IBD diagnosis to IPAA, years, mean ±SD (range)	21.7±12.1 (4–40)	10.0±9.7 (2–30)	4.5±2.6 (1–7)	NA	NA	NA	0.012^†^
Age at IPAA, years, mean ±SD (range)	45.2±13.6 (23–61)	33.2±14.6 (14–57)	32.7±14.9 (20–52)	NA	NA	NA	NS^†^
Pouch age, months, mean ±SD (range)	89.6±75.9 (6–234)	113.7±69.4 (17–220)	96.5±83.5 (10–188)	NA	NA	NA	NS^†^
Ashkenazi origin	6	7	1	4	8	4	NA
Non-Ashkenazi origin	4	4	0	2	2	6	NA
Mixed origin	2	1	3	4	2	1	NA
Never smoker	10	11	3	6	3	8	NA
Past smokers	0	0	1	2	3	1	NA
Current smokers	2	1	0	2	2	2	NA
Body mass index, mean ±SD (range)	26.1±4.5 (19–33)	24.9±5.4 (17–34)	23.1±6.4 (18–33)	23.1±4.8 (18–32)	25.6±8.0 (17–42)	27.7±5.4 (19–36)	NS^†^
Family history of IBD	2/12	5/12	0/4	5/10	0/8	2/11	NA
Steroid exposure prior to IPAA	9	10	4	NA	NA	NA	NA
Anti-TNFs exposure prior to IPAA	0	2	0	NA	NA	NA	NA

SD- standard deviation, NA- not applicable, NS- not significant, IBD- inflammatory bowel disease, IPAA- ileal pouch anal anastomosis*Chi-square test; ^†^Kruskal Wallis Test.

### miRNA Expression in the Ileal Mucosa of Patients with UC Is Comparable to That of NCs

The majority of UC patients who undergo IPAA will develop pouchitis. We therefore sought to determine whether the intact ileum of UC patients has predisposing aberrations in miRNA expression that may expose them to ileal inflammation. To this end, we compared the expression of 1194 miRNAs detected in the biopsies of our entire cohort using NGS analysis. First, we compared miRNA expression in biopsies obtained from the terminal ileum of unoperated UC patients and NC. Importantly, there were no differences in miRNA expression between those groups that met the criteria of both P value (FDR) ≤0.05 and fold change >±2.

### Only Few miRNA Expression Alterations Exist in NP Mucosa

**Fig 1:**
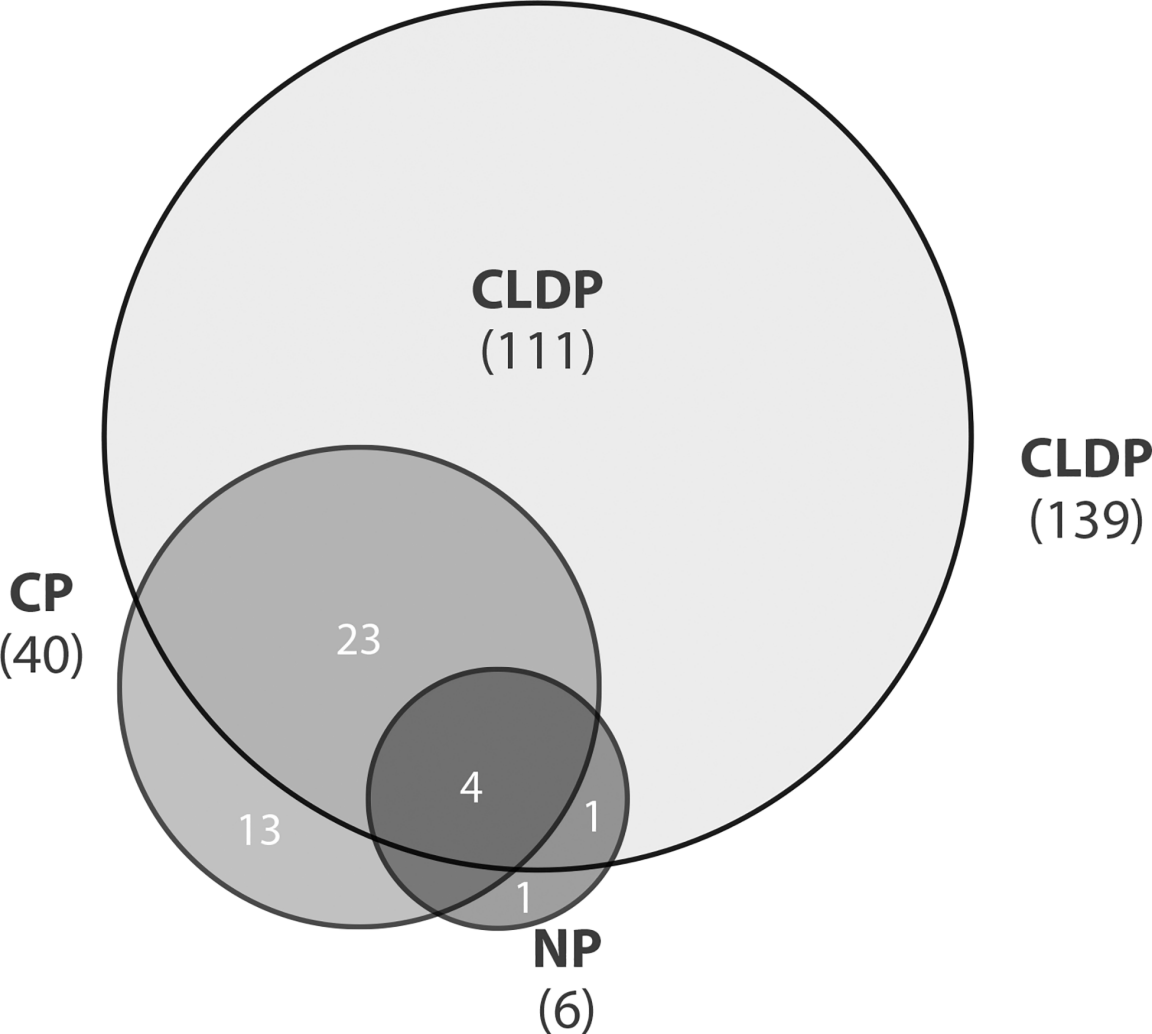
Overlap in altered microRNAs in the three pouch groups. The Venn diagram shows the overlap of microRNA alterations among the pouch groups (normal pouch [NP], chronic pouchitis [CP], and Crohn’s-like disease of the pouch [CLDP]). The numbers in parentheses represent the total number of alterations compared to NC in each group.

Having determined that the terminal ileum of the UC patients was comparable to that of the NC, we next investigated whether the NP pouch mucosa is comparable with the ileal mucosa of NC. Although they had an endoscopically and histologically normal appearing mucosa, six miRNA transcripts of the NP patients were significantly altered compared to the mucosa of the NC (Fig 1, S1A Table).

### Pouch Inflammation Correlates with miRNA Expression Alterations

A total of 40 miRNA expression alterations were detected within the pouches of CP patients compared with NC; 13 (32.5%) of these were unique to CP and the rest were commonly altered in CLDP (Fig 1, S1B Table). Moreover, CLDP patients, who represented the most severe clinical phenotype of pouch complications, had 139 significantly altered miRNAs (Fig 1, S1C Table), 80% of which (111/139) were unique to CLDP. This was approximately 3.5-fold higher compared to the alterations in CP, and 23-fold higher compared to those in NP. Interestingly, four and five out of the six miRNAs alterations detected in NP were also observed in CP and CLDP samples, respectively (Fig 1). Similarly, the magnitude of miRNA alterations in the CLDP group was the largest; mir-371b demonstrated the highest level of alteration, 87 times more than in the NC group. Correspondingly, the largest levels of alteration in the CP and NP groups were found for mir-1910 (23-fold) and mir-148a (3-fold), respectively (S1A–S1C Table).

### Crohn’s Ileitis is Associated with Only a Few miRNA alterations

Only two significant miRNA expression alterations were detected in CD compared to NC patients, none of which were unique. These two altered miRNAs (mir-659 and mir-183) were found in CLDP, and one of them (mir-183) was found in the CP group as well (S1D Table)

### Cluster Analysis Demonstrates an Association Between Pouch Phenotype and miRNA Expression Among IBD patients

**Fig 2:**
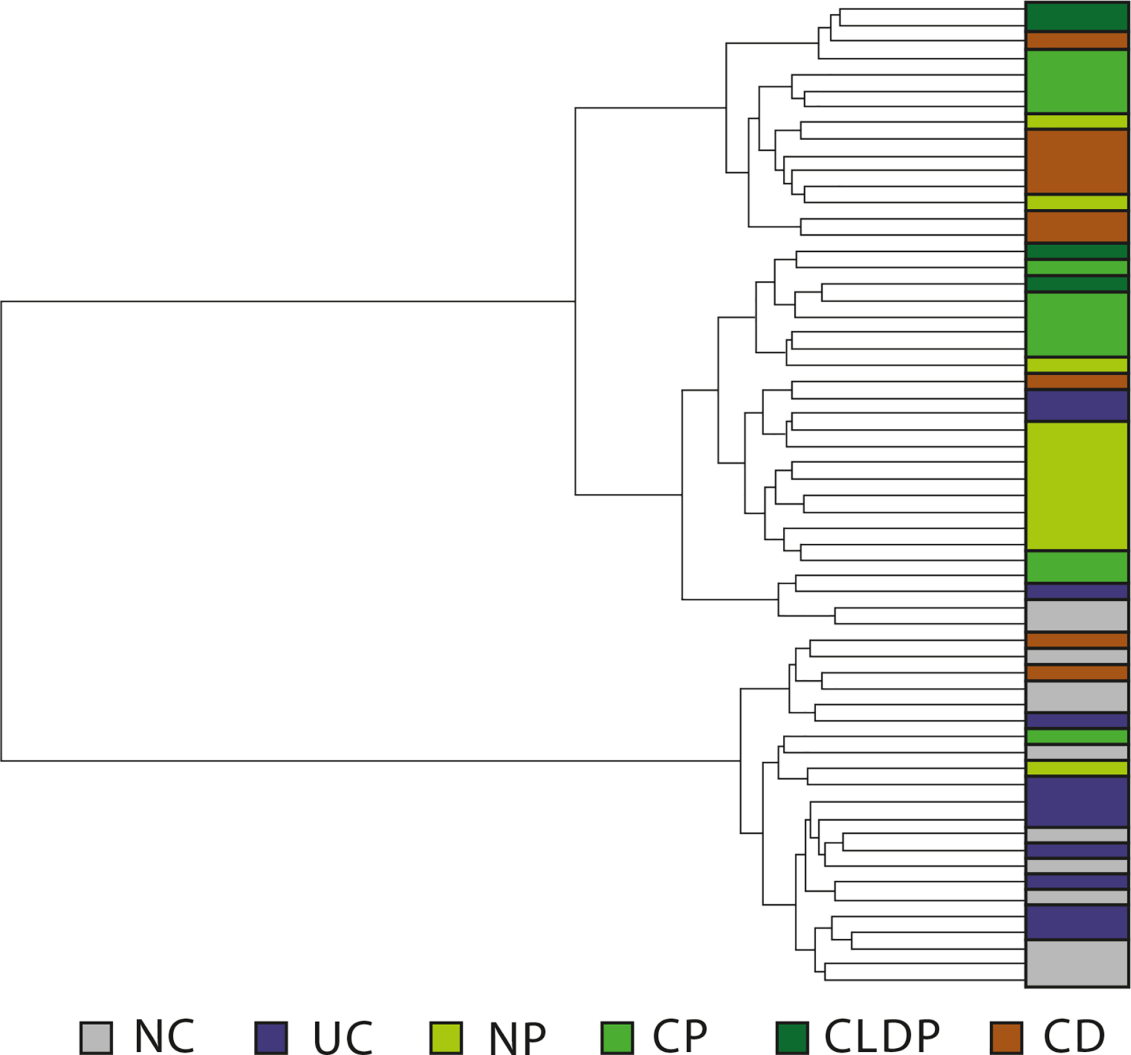
Cluster analysis and a hierarchical tree comparing mucosal microRNA expression alterations in ileal mucosa of different IBD subgroups and normal controls. Cluster analysis and a hierarchical tree comparing microRNA expression alterations in IBD subgroups: normal pouch (NP), chronic pouchitis (CP), Crohn’s-like disease of the pouch (CLDP), unoperated ulcerative colitis (UC), ileal Crohn’s disease (CD), and normal controls (NC). Each bar represents a single individual. The hierarchical tree is represented by black lines.

We next determined whether IBD patients could be clustered according to their miRNA expression profile. During the analysis of the entire expression profile, two main clusters emerged: the first cluster was mainly comprised of the NC and UC groups (81%, 17/21, the “normal ileum” cluster), while the second included all pouch groups and most of the CD group (80%, 8/10, the “pouchitis” cluster). Of note, only 4 samples of NC and UC were clustered within the second cluster, while none of the pouch patients were clustered within the normal ileum cluster (Fig 2).

### The Vast Majority of Altered miRNAs in Pouch Patients Are Up-regulated

Although miRNA expression can be either up- or down-regulated, we were surprised to note that the vast majority of the significantly altered miRNAs showed increased expression compared to levels in NC: 5/6 (83%) in NP, 35/40 (87.5%) in CP and 132/139 (95%).

Given the directionality of the alterations in miRNA expression and the fact that mostly one protein, Dicer, plays a key role in miRNA biosynthesis,[19] one could speculate that alterations in *DICER* expression levels may be one reason for the observed directionality. However, no such alterations were observed in the gene expression data.[18]

Finally, since multiple miRNAs are located within the exons and introns of genes, it might have been possible that once an alteration in mRNA occurs, it drives a concomitant alteration in miRNA(s) located within the same gene. Interestingly, 68/139 of the altered miRNAs in the CLDP group were located within genes (S2 Table); however, only five of these were located within genes whose transcripts were significantly altered. Thus, these findings suggest that alterations in miRNAs in pouchitis occur independently of alterations in mRNA.

### Validation of miRNA Alterations

**Fig 3:**
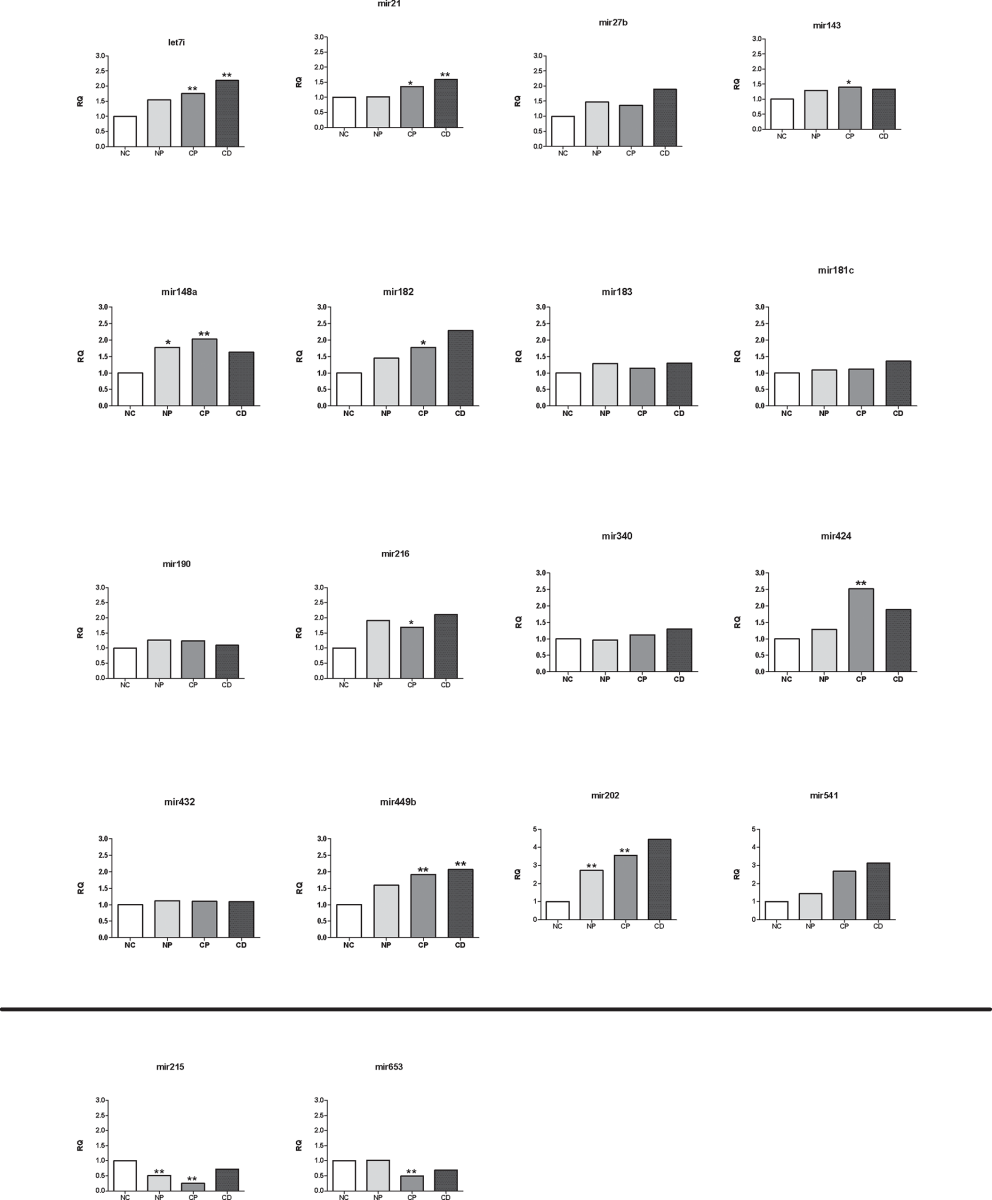
Validation of miRNA alterations. Validation of 18 altered miRNAs obtained from ileal/pouch biopsies from an independent cohort of patients with normal pouch (NP), chronic pouchitis (CP) and Crohn’s-like disease of the pouch (CLDP), and normal controls (NC) using RT-PCR. The average levels of controls were designated as relative quantification (RQ) 1. *P <0.05, **P <0.01.

We next obtained total RNA from ileal/pouch biopsies of an independent cohort in order to validate the results of our NGS miRNA assessments. We tested biopsies from 17 controls (10 NC and 7 unoperated UC patients), 10 CP and 9 CLDP patients, which were grouped into the pouchitis group. Eighteen miRNAs that were altered in the NGS experiment were tested for each group using qRT-PCR. These miRNAs were selected based on specific interest; twelve of these miRNAs were altered by sequencing analysis in all pouch groups, and six miRNAs were altered in both CP and CLDP groups (S1A–S1C Table). Sixteen of these tested miRNAs showed increased expression in the CP group, and two showed decreased expression. We were able to validate 10 of those 16 increased miRNAs (62.5%). Similarly, both down-regulated miRNAs were down-regulated by qRT-PCR as well (Fig 3).

### Inflammatory Conditions Induce Increased miRNA Expression in Intestinal Epithelial Cells

**Fig 4:**
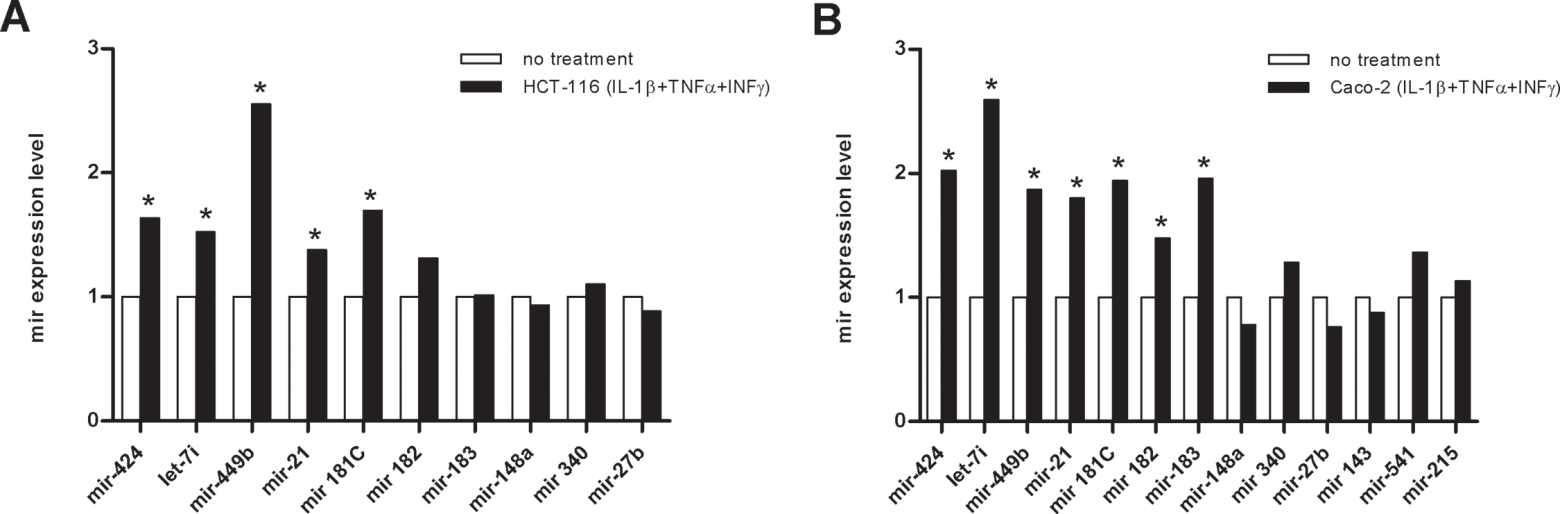
Inflammatory conditions induce increased miRNA expression in intestinal epithelial cells. (A) HCT-116 and (B) Caco2 cells after incubation with pro-inflammatory cytokines. Relative microRNA expression alterations were calculated for each tested microRNA (mir) after treatment with IL-1β, TNF-α, and INF-γ, compared to the expression level of the corresponding untreated cells. *P <0.05.

Given the robust increased expression of miRNAs in pouch inflammation, we tested whether inflammation induced *in vitro* would be similarly accompanied by miRNA up-regulation. Thirteen of the 18 tested miRNAs were expressed in Caco-2 epithelial cells. After incubation of Caco-2 cells with IL-1β, TNF-α, and INF-γ, six of those miRNAs were up-regulated, whereas seven remained unchanged. Similarly, when the experiment was performed using HCT cells that constitutively expressed 10 of the 18 tested miRNAs, an increase in 7/10 microRNAs was observed, while that of three was unchanged. These results suggest that inflammatory conditions induce increased miRNA expression in intestinal epithelial cells (Fig 4).

## Discussion

Gene regulation is known to play a major role in physiological and pathological conditions. We previously showed that inflammation in IBD is associated with multiple alterations in mRNA expression, correlating with clinical phenotype.[18] As one of the most prominent mRNA regulatory mechanisms is the miRNA machinery, its role in IBD has been previously evaluated. So far, only limited number of miRNAs have been associated with IBD. Wu et al.[26] detected five miRNAs that were differentially expressed in active CD colitis, and five miRNAs that were differentially expressed in active CD ileitis. Similarly, 11 miRNAs were differentially expressed in active UC.[25] Interestingly, Fasseu et al.[36] detected alterations in miRNA expression even in non-active IBD. Other studies have shown that specific miRNAs may serve as biomarkers for differentiating between CD and UC in undefined cases,[27, 37] serve as disease severity markers,[38] or even serve for IBD diagnosis.[39]

In the current study, we used massive parallel sequencing to detect miRNA expression in IBD pouch patients. For the first time, we showed that a large number of miRNAs (153) were significantly altered in IBD patients with a pouch (NP: 6, CP: 40, CLDP: 139, Fig 1). This was demonstrated by using a conservative definition for alteration, i.e., a >2-fold change and a corrected P value <0.05. miRNA profiles could be used for clustering of pouch patients into subgroups (Fig 2), as was previously shown by us for mRNA profiles. Interestingly, while only six miRNAs were altered in NP, based on the overall miRNA profile, the great majority of the NP patients clustered within the pouchitis cluster (Fig 2). This implies that a normal-appearing pouch already harbors molecular alterations that may represent early processes in the development of inflammation, or, alternatively, that subtle inflammation is present even though it is not detected endoscopically or microscopically. This latter implication is supported by the report by Fasseu et al.,[36] who demonstrated the presence of miRNA alterations in inactive IBD. This pattern of altered miRNA occurring in NP patients is in accordance with our previous observation that mRNA altered expression existed even in the normal-appearing proximal small bowel mucosa of patients with pouchitis.[31]

Given the significant differences in IBD duration and the time between IBD diagnosis and surgery in the different pouch phenotypes (Table 1), it is possible that disease duration and/or pouch age by themselves have some impact on miRNA expression. Given the sample size and the multiple phenotypes, these hypotheses could not be tested in the current study.

Most, but not all, patients with CD clustered with patients who had an inflamed pouch (the CP and CLDP groups), in agreement with the heterogeneous nature of CD. This heterogeneity may also be the reason for the lack of detection of multiple miRNA alterations by previous studies, since those focused mainly on CD. In contrast to only two alterations in miRNA expression detected in CD in our study, dozens of miRNAs alterations were detected in the different pouch phenotypes. Moreover, in contrast to the heterogeneity of CD, patients with a pouch could be better defined into phenotypic groups, thus decreasing variability and consequently increasing statistical power. This defined phenotype makes pouchitis and especially its most severe phenotype, CLDP, an appropriate model to study intestinal inflammation in IBD.

The relatively large number of altered miRNAs that were detected using our approach enabled us to delineate the directionality of miRNA alterations for the first time. While the exact reason for this robust up-regulation of miRNA remains unclear, our *in silico* data suggest that the increased miRNA expression is not the result of increased expression of mRNAs containing miRNAs loci, or secondary to alterations in the expression of *DICER*.

In summary, using pouchitis as a model for the development of IBD, we noted multiple miRNA expression alterations that were associated with clinical phenotypes. Interestingly, the vast majority of altered miRNAs in patients after pouch surgery were up-regulated, possibly due to a pro-inflammatory state. Globally increased miRNA expression in IBD may have a role in the down-regulation of mRNA transcripts. The massive involvement of miRNA in IBD inflammation, as demonstrated in patients with a pouch, may contribute to the understanding of the complexity of IBD. While the contribution of miRNA alterations to disease pathophysiology is still unclear, these changes may serve as a biomarker of disease phenotype. Furthermore, it is possible that the different miRNA profiles that correlate with phenotype will be modified by therapy. In such cases, miRNA profiling may be used to monitor treatment effects. Further longitudinal studies are required in order to test this possibility. Overall, better delineation of the involvement of miRNA in the inflammatory process in IBD may open up an opportunity to understand and consequently modify disease mechanisms.

## Supporting Information

S1 Table miRNA expression alterations in IBD. (XLS)

S2 Table Genomic location of altered miRNAs. (XLS)
