# Correction: MicroRNAs Expression in the Ileal Pouch of Patients with Ulcerative Colitis Is Robustly Up-Regulated and Correlates with Disease Phenotypes

**DOI:** 10.1371/journal.pone.0165220

**Published:** 2016-10-18

**Authors:** Shay Ben-Shachar, Henit Yanai, Hadas Sherman Horev, Hofit Elad, Liran Baram, Ofer Isakov, Hagit Tulchinsky, Metsada Pasmanik-Chor, Noam Shomron, Iris Dotan

The name of the sixth author is incorrect. The correct name should be: Ofer Isakov. The correct citation should be: Ben-Shachar S, Yanai H, Sherman Horev H, Elad H, Baram L, Isakov O, et al. (2016) MicroRNAs Expression in the Ileal Pouch of Patients with Ulcerative Colitis Is Robustly Up-Regulated and Correlates with Disease Phenotypes. PLoS ONE 11(8): e0159956. doi:10.1371/journal.pone.0159956
